# Role of Cardio-Specific Micro-Ribonucleic Acids and Correlation with Cardiac Biomarkers in Acute Coronary Syndrome: A Comprehensive Systematic Review

**DOI:** 10.7759/cureus.5878

**Published:** 2019-10-09

**Authors:** Raja S Mushtaque, Sajid Hameed, Rabia Mushtaque, Muhammad Idrees, Farah Siraj

**Affiliations:** 1 Internal Medicine, Jinnah Postgraduate Medical Center, Karachi, PAK; 2 Neurology, Aga Khan University, Karachi, PAK; 3 Cardiology, National Institute of Cardiovascular Diseases (NICVD), Karachi, PAK; 4 Internal Medicine, Bassett Medical Center, Cooperstown, USA; 5 Internal Medicine, Chandka Medical College Hospital, Larkana, PAK

**Keywords:** acute coronary syndrome, microrna, mirna, acute myocardial ischemia, cardiac biomarkers, acs

## Abstract

Acute coronary syndrome (ACS) is an acute and severe manifestation of coronary artery disease (CAD); thus, timely diagnosis can save a life. Commonly, cardiac troponin T (CTnT), cardiac troponin I (CTnI) or creatine kinase muscle/brain subtype (CK-MB) have been used as cardiac biomarkers to assess ACS with certain limitations, such as increased time to rise for diagnosis and increased levels in the patients with chronic kidney disease (CKD). Recently, micro-ribonucleic acids (miRNAs) have become potential candidates as biomarkers for cardiac ischemia due to their remarkable stability and reproducibility. Certain miRNAs, for instance, miR-1, miR-133a/b, miR-208a/b, and miR-499a, strongly increase in the serum or plasma of patients with acute cardiac ischemia, making them as cardio-specific miRNAs and prospective biomarkers in ACS. This literature review gives enlightenment about the regulation of cardio-specific miRNA in acute myocardial ischemia (AMI) and correlation with common cardiac biomarkers and time at which they increase in the blood.

## Introduction and background

Coronary artery disease (CAD), also known as ischemic heart disease (IHD) is the most common cardiovascular disease [1]. Acute coronary syndrome (ACS) is associated with rupture of an atherosclerotic plaque and partial or complete thrombosis of the infarct-related artery; ACS refers to a spectrum of clinical presentations ranging from acute myocardial infarction (AMI) either ST-segment elevation myocardial infarction (STEMI) or non-ST-segment elevation myocardial infarction (NSTEMI) and unstable angina [2]. Commonly, cardiac troponin T (CTnT), cardiac troponin I (CTnI) or creatine kinase brain/muscle subtype (CK-MB) have been used as biomarkers to diagnose and assess the prognosis of AMI but have certain limitations. CTnT or CTnI begins to rise within three to four hours, and CK-MB first appears four to six hours after the onset of myocardial injury. These biomarkers are well known to be increased in patients with chronic kidney disease (CKD) and can be misleading even in the absence of clinically suspected myocardial ischemia [3]. Thus, there is still a clinical need for a novel biomarker, which can reliably rule in or rule out AMI immediately. Micro-ribonucleic acids (miRNAs) seem to be a promising candidate for such a novel biomarker for the early diagnosis of AMI.

miRNAs are short (~22 nucleotides) endogenous RNAs. More than 2000 miRNAs have been discovered in humans, and it is believed that they collectively regulate one-third of the genes of our genome and many of them have already been implicated in common human disorders [4]. The remarkable stability of miRNAs in blood and urine has also made them interesting candidates as biomarkers for various pathological conditions. Certain miRNAs, for instance, miR-1, miR-133a/b, miR-208a/b, and miR-499a, were reported many times as being strongly increased in the serum or plasma of patients with AMI [5]. This review article summarizes available literature about cardio-specific miRNA and their role in ACS and their correlation with traditional cardiac biomarkers. It also evaluates the potential of miRNAs as useful diagnostic biomarkers for early diagnosis of AMI.

## Review

Materials, methods, and results

**Figure 1:**
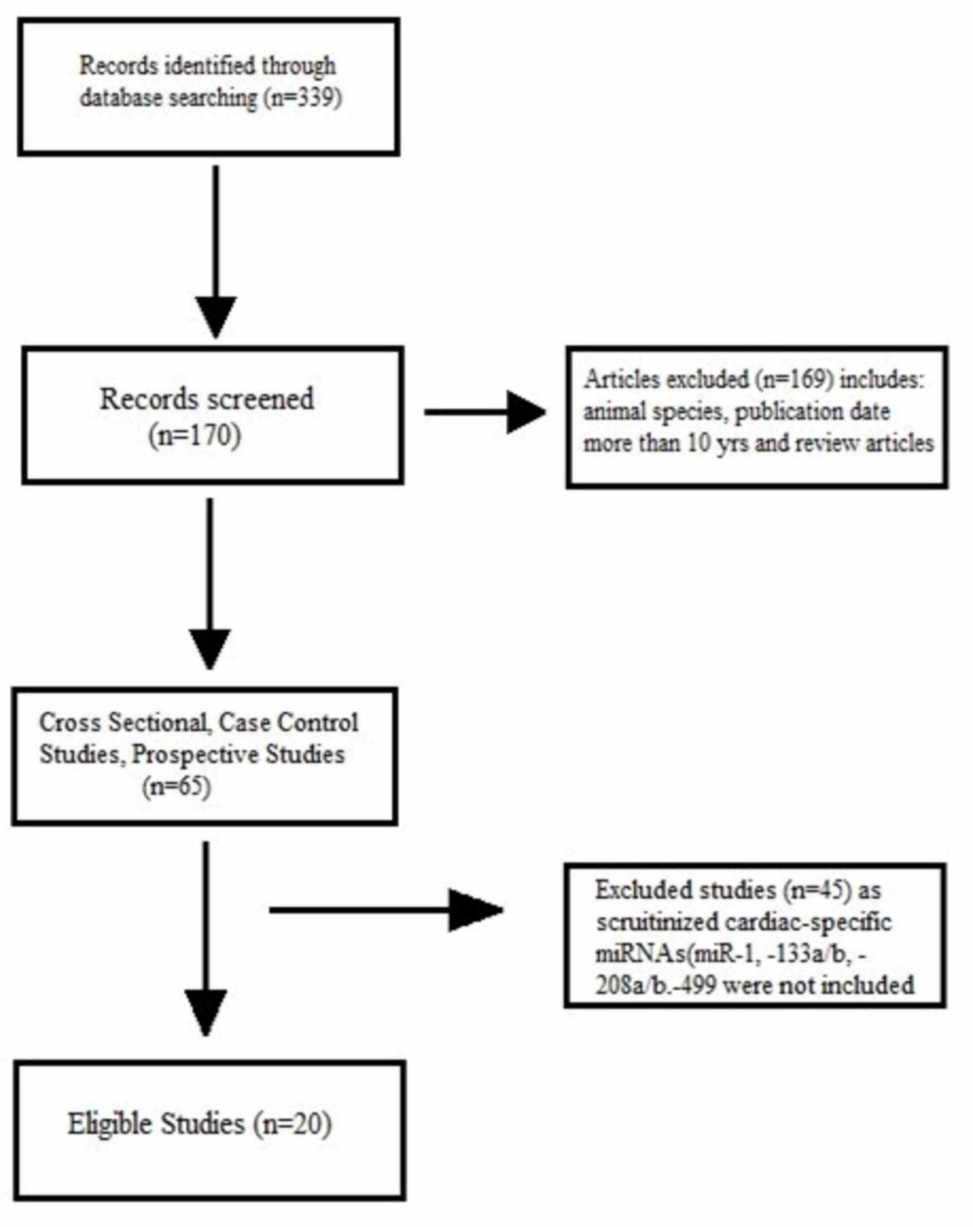
Material and Methods: Criteria for the selection of articles. miRNA/miR = micro-ribonucleic acid

The literature search was done utilizing online libraries: Medline database, Excerpta Medica database, and Cochrane library (PubMed/EMBASE/Cochrane library). The following keywords and medical subject headings (MeSH) terms were used: “Acute myocardial infarction,” “AMI,” “Acute coronary syndrome,” “ACS,” “microRNA,” “miRNA” and “miR.” We included the articles that were published in the English language, contain human specimens, carried out from January 2010 onwards, and contain patients with acute coronary syndrome (ACS) as an outcome. We excluded studies with congenital heart disease or valvular heart disease. We only included the case-control studies in our article, which were 20 in number (Figure 1). We also examined the references of all studies from the initial search for additional references.

A total of 20 clinical studies were included in this review after a thorough analysis of the literature. Overall, there were 3560 subjects. Eight out of 20 studies included were done in China, while two studies each were from Japan, Luxembourg, Sweden, Italy, and one study each from Germany, Poland, Netherlands, and Greece (Table 1). In 13 out of 20 studies, the source of extraction of microRNA was plasma. In six studies, the source of extraction of microRNA was serum, and only one study (Wang et al., 2011) used both whole blood and plasma as the source of microRNA extraction [6].

**Table 1:** Characteristics of the selected studies AMI: Acute myocardial infarction; CHD: Chronic heart disease; ACS: Acute coronary syndrome; UAP: Unstable angina pectoris; AP: Angina pectoris; CHF: Chronic heart failure; NSTEMI: Non ST elevation myocardial infarction; STEMI: ST elevation myocardial infarction; UA: Unstable angina; CAD: Coronary artery disease

Serial no.	Author, Year	Study Population	Country	Type of study	Outcome
1.	Wang et al, 2010, [7]	AMI (n=33); Non AMI (n=33)	China	Case Control	ACS
2.	Adachi et al, 2010, [8]	AMI (n=9); UAP (n= 5); CHF III (n=9); CHF II (n=6); Control (n=10)	Japan	Case Control	ACS
3.	Corsten et al, 2010, [9]	AMI (n=32); Control (n=36)	Luxembourg	Case Control	ACS
4.	D’Alessandra et al, 2010, [10]	AMI (n=33); Control (n=17)	Italy	Case Control	ACS
5.	Zhang et al, 2010, [11]	AMI (n=93); Control (n=66)	China	Case Control	ACS
6.	Cheng et al, 2010, [12]	AMI (n=31); Control (n=20)	China	Case Control	ACS
7.	Wang et al, 2011, [6]	AMI (n=51); Control (n=28)	China	Case Control	ACS
8.	Widera et al,2011, [13]	STEMI (n=196); NSTEMI (n=131); Unstable angina (n=117)	Germany	Case Control	ACS
9.	Kuwabara et al, 2011, [14]	ACS (n=29); Control (n=42)	Japan	Case Control	ACS
10.	Gidlof et al, 2011, [15]	AMI (n=25); Control (n=11)	Sweden	Case Control	ACS
11.	Devaux et al, 2012, [16]	STEMI (n=397); NSTEMI (n=113); Control (n=87)	Luxembourg	Case Control	ACS
12.	Olivieri et al, 2012, [17]	NSTEMI (n=92); CHF (n=81); Control (n=99)	Italy	Case Control	ACS
13.	Oerleman et al, 2012, [18]	ACS (n=106); Non ACS (n=226)	Netherlands	Case Control	ACS
14.	Li et al, 2013, [19]	AMI (n=117); AP (n=182); Control (n=100)	China	Case Control	ACS
15.	Gidlof et al, 2013, [20]	AMI (n=319); Non AMI (n=88)	Sweden	Case Control	ACS
16.	Li YQ et al, 2013, [21]	AMI (n=67); Control (n=32)	China	Case Control	ACS
17.	Chen et al, 2014, [22]	AMI (n=53); UA (n=20); Control (n=30)	China	Case Control	ACS
18.	Zhao et al, 2015, [23]	AMI (n=59); Control (n=60)	China	Case Control	ACS
19.	Białek et al, 2015, [24]	STEMI (n=19); Stable CAD (n=12); Control (n=8)	Poland	Case Control	ACS
20.	Agiannitopoulos et al, 2018, [25]	AMI (n=80); Control (n=50)	Greece	Case Control	ACS

The common technique used to extract microRNA in all studies was a real-time polymerase chain reaction (RT-PCR). Our systematic review focused on cardiac-enriched microRNAs (e.g., miR-1, miR-133a, and miR-208b, miR-499). These were consistently found to be rapidly up-regulated in the sample sources after myocardial necrosis in a majority of available studies. The majority of the studies also mentioned the time of blood sampling after symptoms started.

Four studies analyzed four miRNA, miR-1, miR-133a/b, miR-208a/b, and miR-499, collectively [7,13,15,21]. In a study by Wang et al., all four microRNA levels were up-regulated, significantly higher than control (p <0.01), and positively correlated with cTnI while the mean timing of blood sampling was 4.8+3.5 hours [7]. miR-208a displayed a higher diagnostic significance for AMI, with the area-under-curve (AUC) of 0.965 (95% CI, 0.920-1.000), than other miRNAs but was lower than CTnI- AUC of 0.987 (95% Cl, 0.966-1.000). They also noticed that in three AMI patients, miR-208a levels became detectable within 1-4 hours of chest pain, when the CTnI level was still detected below the cut-off value. In Widera et al., patients with NSTEMI or STEMI presented with higher levels of miR-1, miR133a, and miR-208b compared with patients with unstable angina [13]. In a multiple linear regression analysis that included clinical variables and CTnT, miR-1, miR-133a, miR-133b, and miR-208b were independently associated with CTnT levels (all p <0.001). In Gidlof et al., all miRNA levels were substantially higher than those from healthy people (p < 0.001) [15]. miR-208b positively correlated with cTnT and negatively correlated with ejection fraction (EF) while other miRNAs did not correlate with either CTnT or EF [15]. The blood samples were collected at 24 hours, 48 hours, and 72 hours levels. The circulating miR-1, miR-133a, miR-208b, and miR-499-5p were elevated within 12 hours of the onset of symptoms in STEMI patients. In Li YQ et al., the levels of miR-1, -133a, -208b, and -499 were markedly increased in plasma samples gathered within 12 hours of the onset of AMI, but the four up-regulated miRNAs were not superior to CTnT for the diagnosis of AMI (p ˃0.05) [21].

Three studies (Corsten et al., Devaux et al., and Agiannitopoulos et al.) analyzed two microRNAs, miR-208b and miR-499, together and found both up-regulated [9,16,25]. In Corsten et al., miR-208b was 1600-fold up-regulated (p <0.005), while miR-499 was 100-fold higher (p <0.0005), and they correlated significantly with CTnT (p=0.0005 and p=0.0001, respectively) while the time of sampling was < 12 hours. In Devaux et al., both miR-208b and miR-499 were (a) higher in patients with AMI than in controls (p <0.0001), (b) higher in patients with STEMI than NSTEMI (p <0.0001), and (c) significantly correlated with CK and CTnT (p <10^-9^) but were inversely correlated to the EF, with correlation coefficients of -0.18 (p <0.0008) and -0.17 (p <0.001), respectively [16]. In Agiannitopoulos et al., both miRNAs were significantly higher in patients with AMI than control (p <0.0001) and correlated with CTnT (p <0.0001) [25].

Three studies (Adachi et al., Chen et al., and Zhao et al.) analyzed miR-499 individually and found it up-regulated [8,22,23]. Adachi et al. found miR-499 was significantly higher in the AMI group than the other groups (p <0.0001), and it correlated with CK-MB; the time of blood sampling was within 48 hours after onset of chest pain [8]. The peak plasma miR-499 concentration occurred between six hours and 12 hours [8]. In Chen et al., the time of blood sampling was at 0 hours, 12 hours, 24 hours, three days, and seven days after onset of symptoms [22]. The average duration between the onset of chest pain and arrival at the emergency room was 4.46±3.36 hours. The relative level of plasma miR-499 in 53 patients with AMI (5.12±2.29) was significantly higher than that in unstable angina (UA) group (2.75±1.39), and healthy control group (0.50±0.35); the differences were statistically significant (p <0.01) and positively-correlated with CTnI (r=0.384, p <0.01) and CK-MB (r=0.402, p <0.01) [22]. In Zhao et al., miRNA-499 in AMI was significantly higher than in controls (p <0.05) [23]. mRNA-499 could be detected in the serum three hours after the onset of AMI, reached a peak value after 12 hours, and gradually declined after 15 hours [23]. miR-499 in the diagnosis of AMI was still lower than those of CTnI [CTnI-AUC=0.971 (95% CI, 0.951-1.000), miR-499-AUC=0.915 (95%CI, 0.826-1.000)] [23].

Two studies (Zhang et al. and Cheng et al.) investigated miR-1 and found it up-regulated [11,12]. In Zhang et al., the miR-1 level was significantly increased and positively correlated with cardiac troponin [11]. In Cheng et al., miR-1 was higher than controls (p <0.05) and positively correlated with CK-MB levels (r=0.68; p <0.05) while the mean-time of blood sampling was 8.5±3.82 hours [12].

D’Alessandra et al. found that miR-1, -133a, -133b, and -499-5p were up-regulated, and miR-122 and 375 were down-regulated [10]. miRNAs levels were significantly changed in the AMI group vs. control (p <0.01), and miRNAs were positively correlated with CTnI (p <0.01). The mean blood sampling was done at 517+309 min after the onset of symptoms. miR-1, -133a, and -133b plasma levels were already at their peak at T0, i.e., at a time point very close to the peak increase in CTnI. In contrast, miR-499-5p exhibited a slower time course and peaked after CTnI. At the end of the 3-day time course, miR-1, -133a, -133b, and -499-5p had returned close to their control levels.

Wang et al. evaluated miR-133 and miR-328, and both were up-regulated [6]. The increase in miR-133 (4.4-fold) in patients with AMI vs. control (p=0.006) in whole blood samples was comparable to plasma. The miR-328 levels in plasma and whole blood of AMI patients were markedly increased, by 10.9-fold and 16.1-fold respectively, as compared to control (p=0.033 and p <0.001). The samples were obtained at 5.24±1.38 hours after AMI (T0). Cardiac troponin I was significantly increased at T0, remained increased until 20 hours, and was finally restored to the normal value seven days after T0. In contrast, both miR-133 and miR-328 levels in plasma or whole blood samples were already at their peak values at T0. The elevated circulating miR-133 and miR-328 were decreased 20 hours after T0 and returned to the control levels at seven days after T0. However, the miR-133 and miR-328 exhibited faster peaks than CTnI. There was a positive correlation between circulating miR-133 or miR-328 levels and CTnI.

Kuwabara et al. analyzed miR-1 and miR-133a, and both were up-regulated (p <0.0005 and p <0.0001, respectively) [14]. Both miRNAs were positively correlated with CTnT; miR-1 (p <0.005) and miR-133a (p <0.0001).

Oerlemans et al. investigated miR-1, miR-208a, miR-499, miR-21 and miR-146a and all were up-regulated: miR-1 (OR, 1.44; 95% CI, 1.19-1.73); miR-208a (OR, 1.12; 95%CI, 0.95-1.35); miR-499 (OR, 1.38; 95% CI, 1.19-1.61); miR-21 (OR, 1.34; 95% CI, 1.15-1.55); miR-146a (OR, 1.06; 95%CI, 0.97-1.15) [18]. They found miR-1+miR-499+miR-21 superior to CTnT (p<0.001).

Gidlöf et al. found that miR-1, miR-208b, and miR-499-5p were up-regulated [20]. miR-208b and miR-499-5p were significantly higher in both NSTEMI and STEMI patients compared to non-MI patients (p < 0.001) but below the current gold standard cardiac marker, CTnT. The mean time for blood sampling was 38.4 hours [20].

Olivieri et al. found that plasma levels of miR-1, -21, -133a, -208a, -423-5p and -499-5p were up-regulated [17]. However, miR-499-5p exhibited the highest increase compared to other miRNAs; NSTEMI vs. control (p <0.001); NSTEMI vs. congestive heart failure (CHF) (p <0.05) and CHF vs. control (p <0.05). In the total population, including NSTEMI, acute CHF and control, miR-499-5p was significantly correlated with CTnT (p <0.001) [17].

Li et al. found up-regulation of six miRNAs compared to control; miR-1, miR-223 and miR-499 (p <0.05), and miR-134, miR-186 and miR-208 (p <0.001) [19]. Although among these six miRNAs, miR-208 and miR-499 were elevated higher in angina pectoris (AP) cases than in AMI cases. The AUC values of the six-serum miRNAs signature (AUC, 0.830; 95% CI, 0.751-0.910) were higher than those of CTnT (AUC, 0.768; 95% CI, 0.672-0.864) and CK-MB (AUC, 0.709; 95% CI, 0.606-0.812).

In a study by Bialek et al., miRNA-208a was increased in STEMI patients at the time of admission and nearly undetectable in CAD patients and controls (p <0.001) [24]. miRNA-208a levels strongly correlated with CTnI and CK-MB mass; CTnT (p <0.05) and CK-MB mass (p <0.05). A significant increase in the level of plasma miRNA-208a on admission (time 0) in patients with STEMI was observed. The plasma concentration of miRNA-208a increased within the first three hours after the presentation and reminded increased until 12 hours. It is noteworthy that the concentrations of both cardiac biomarkers (CTnI and CK-MB mass) were below the cut off for MI at the time of admission. They increased later, peaking at six hours after admission, and remained elevated during observation up to 48 hours.

The results of all the 20 studies are summarized in Table 2.

**Table 2:** Summary of the results of all 20 studies miR: microRNA; qRT: Quantitative real time; PCR: Polymerase chain reaction; CTnI: Cardiac troponin I; CK-MB: Creatine kinase-muscle/brain; CTnT: Cardiac Troponin T; NSTEMI: Non ST elevation myocardial infarction; STEMI: ST elevation myocardial infarction; hsTnT: High sensitive troponin T; EF: Ejection fraction; RT-PCR: Real time polymerase chain reaction; CHF: Congestive heart failure; CTR: Control; AMI: Acute myocardial infarction; UA: Unstable angina

Serial No.	Author & Year	miRNA Regulation	Source	Analysis Technique	Time of Blood Sampling	Study Results	Correlation with Biomarkers
1.	Wang et al, 2010, [7]	Up-regulated: miR-1, miR-133a, miR-499, miR-208a	Plasma	qRT-PCR	4.8±3.5 h	miRNA levels were substantially higher than those from control P< 0.01.	miRNAs were correlated with cTnI.
2.	Adachi et al, 2010, [8]	Up-regulated: miR-499	Plasma	qRT-PCR	48hrs	miR-499 values in the AM group were significantly higher than those of the other groups (P< 0.0001).	CK-MB
3.	Corsten, et al, 2010, [9]	Up-regulated: miR-208a, miR-499	Plasma	PCR	<12hrs	miR-208b and -499 were highly elevated (P<0.005 and (P<0.0005) in AMI patients, respectively, as compared with control subjects.	miR-208b& miR-499 correlated significantly with CTnT.
4.	D’Alessandra et al, 2010, [10]	Up-regulated: miR-1, miR-133a/b, miR-499-5p; Down-regulated: miR-122, miR-375	Plasma	qRT-PCR	517+309 min	miRNAs levels were significantly changed in AMI group vs. control: p<0.01.	Positively correlated with CTnI p<0.01 vs. control.
5.	Zhang et al, 2010, [11]	Up-regulated: miR-1	Plasma	PCR	Not given	miRNA levels were significantly increased.	Positive correlation with cardiac troponin.
6.	Cheng et al, 2010, [12]	Up-regulated: miR-1	Serum	qRT-PCR	8.5±3.82 h.	miR-1 was higher in AMI patients than in control group P< 0.05.	miR-1 &CK-MB were positive correlated (r=0.68; p<0.05).
7.	Wang et al, 2011, [6]	Up-regulated: miR-133, miR-328	Whole blood Plasma	RT-PCR	5.24 ± 1.38 hrs & 20 hours & 7 days	miR-133 levels in plasma from AMI patients were increased compared with control group (p=0.006). miR-328 levels in plasma and whole blood of AMI patients were markedly increased compared to those in control subjects (p=0.033 and p<0.001).	Correlated with CTnI
8.	Widera et al, 2011, [13]	Up-regulated: miR-1, miR-133a; Not Significant: miR-133b, miR-208a, miR-208b, miR-499	Plasma	qRT-PCR	Not given	Patients with NSTEMI or STEMI presented with higher levels of miR-1, miR133a, and miR-208b compared with patients with unstable angina. miR P=0.001.	miR 133a P<0.001 miR-1, miR-133a, miR-133b & miR-208b were independently associated with hsTnT levels (p<0.001).
9.	Kuwabara et al, 2011, [14]	Up-regulated: miR-1, miR-133a	Serum	qRT-PCR	Not given	miR-1 p<0.0005, miR-133a p	miRNAs were positive correlated with cTnT.
10.	Gidlof et al, 2011, [15]	Up-regulated: miR-1, miR-133a, miR-208b, miR-499-5p	Plasma	RT-PCR	24hrs, 48hrs, 72hrs	miR-1 (p<0.01); miR-133a (p<0.01) miR-208b (p<0.001) miR-499-5p (p<0.01) Compared to control group.	cTnT positively correlated with miR-208b (p = 0.01, r 2 =0.25);EF negatively correlated with miR-208b (p = 0.01, r 2 = 0.32).
11.	Devaux et al, 2012, [16]	Up-regulated: miR-208b, miR-499	Plasma	RT-PCR	Not given	Both miRNAs were higher in MI patients (p<0.001).	Correlation between miRNAs and CK & CTnT were highly significant P<10¯ 9. miRNAs were inversely correlated to the EF.
12.	Olivieri et al, 2012, [17]	Up-regulated: miR-1, miR-21, miR-133a, miR-423-5p, miR-499-5p	Plasma	qRT-PCR	Not given	NSTEMI versus control p<0.05; NSTEMI versus CHF p<0.05; CHF versus CTR p<0.05	miR-499-5p and cTnT were positively correlated (p<0.001) in the total population as well as in NSTEMI patients.
13.	Oerleman et al,2012, [18]	Up-regulated: miR-1, miR-208a, miR-499, miR-21, miR-146a	Serum	RT-PCR	Not given	Circulating levels of all miRNAs were higher in patients with ACS. Furthermore, circulating levels of miR-21 and miR-146a were markedly elevated in ACS patients as well (p<0.001).	miR-1+miR-499+miR-21: (miRNA combine assay) Superior to hs-CTnT.
14.	Li et al, 2013, [19]	Up-regulated: miR-1, miR-134, miR-186, miR-208, miR-223, miR-499	Serum	RT-PCR	Not given	Serum levels of the six miRNAs were increased in AMI than control subjects: miR-1, miR-223, and miR-499: P<0.05; miR-134, miR-186, miR-208: P<0.001.	Correlated to CTnT and CKMB.
15.	Gidlof et al, 2013, [20]	Up-regulated: miR-1, miR-208b, miR-499-5p	Plasma	qRT-PCR	Mean time To sample:38.4 hours	miR-1 was increased (p<0.01), miR-133a (p<0.01), miR-208b (p <0.001), and miR-499-5p (p< 0.01) as compared to healthy controls.	miR-208b and -499-5p were strongly correlated with TnT but the accuracy was well below that of Troponin T. miR-1 was weakly correlated with TnT.
16.	Li YQ et al, 2013, [21]	Up-regulated: miR-1, miR-133a, miR-208b, miR-499	Plasma	qRT-PCR	Within 12hrs and Day	14 All miRNAs were significantly higher in AMI patients (p<0.001) than in healthy volunteers.	Although these miRNAs and cTnT were positively correlated. But none of the four circulating miRNAs were superior to cTnT for the early diagnosis of AMI (P˃0.05).
17.	Chen et al, 2014,[22]	Up-regulated: miR-499	Plasma	qRT-PCR	0 h, 12 h, 24 h, 72 h, and 7 d after the onset of AMI.	miR-499 levels were significantly higher in AMI patients than in the UA and controls immediately (P<0.01).	miR-499 positively-correlated with cTnI(P<0.01) and CK-MB (P<0.01).
18.	Zhao et al, 2015, [23]	Up-regulated: miR-499	Plasma	qRT-PCR	3h, 12h and 15h	miRNA-499 in AMI was significantly higher than in controls (P < 0.05).	The specificity and sensitivity of microRNA-499 in the diagnosis of AMI were still lower than those of cTnI.
19.	Białek et al, 2015, [24]	Up-regulated: miR-208a	Serum	qPCR	0h, 3h, 6h, 12, 24h & 48hrs	miR-208a was increased in STEMI patients p < 0.001.	miR-208a was Correlated with CTnT and CK-MB-mass.
20.	Agiannitopoulos, et al, 2018, [25]	Up-regulated: miR-208b, miR-499	Plasma	PCR	Not given	miR-208b: p<0.0001, miR-499: p<0.0001, As compared to controls.	Both miRNAs were correlated with CTnT.

Discussion

Recently, there is a huge emphasis on the importance of miRNAs in regulating apoptosis, necrosis, and autophagy in cardiomyocytes, which play a decisive role in myocardial infarction [26]. Studies have also explored the fact that miRNAs are leaked from the heart into the circulation after myocardial injury, during which their expression is elevated and dynamic [27,28]. Thus, the circulating miRNAs in the blood have recently emerged as potential biomarkers for the diagnosis and/or prognosis of AMI due to their stability, specificity, and reproducibility.

Our article summarizes 20 articles comparing miRNAs in patients with ACS. The criteria for ACS/AMI patients’ enrolment in all studies were standardized as per international definitions of STEMI/NSTEMI or unstable angina (UA) [29]. This literature review shows that there is an up-regulation of investigated cardiac-specific miRNAs (miR-1, -133, -208, and -499) in collective or in an isolated manner in all studies while in one study there is down-regulation of two miRNA, -122 and -375 [10]. Besides these miRNAs, few other miRNAs were also found to be up-regulated: miR-328 in Wang et al.; miR-423-5p in Oliveri et al.; miR-21 in Oerleman et al.; and miR-223, -134 and -186 in Li C et al. [6,17-19]. The source of extraction of microRNA was plasma, serum, and whole blood (Table 2). Circulating miRNAs are stable. The common technique used to quantify microRNA was the real-time PCR assay in all studies in our review (Table 2).

Wang et al. revealed for the first time that monitoring the plasma levels of miR-208a could also be applied in the clinical diagnosis of AMI among the four miRNAs investigated [7]. miR-208a had higher sensitivity and specificity for diagnosing AMI, and it was easily detected in AMI patients within four hours of the onset of symptoms, but still, the diagnostic significance was lower than CTnI. No significant difference in age and sex was observed among the three groups.

Adachi et al. showed plasma miR-499 concentrations were elevated in AMI within 48 hours [8]. Peak concentration was observed between six hours and 12 hours and became undetectable before hospital discharge [8]. The up-regulated miR-499 correlated with CK-MB.

Corsten et al. found a significant increase in miR-208b and miR-499, positively correlating with CTnT and CTnI [9]. Plasma microRNA levels were not affected by a wide range of clinical confounders, including age, sex, body mass index, kidney function, systolic blood pressure, and white blood cell count.

D’Alessandra et al. exhibited increased miR-1, -133a, 133b, and 499-5p & decreased miR-122 and 375 and a positive correlation with CTnI [10]. It is noteworthy that the decrease in circulating miR-122 and -375 has not been reported in any other medical condition examined to date, neither in humans nor in the animal models of human diseases.

Zhang et al. showed a significant increase in miR-1 in AMI patients with a positive correlation with cardiac troponins [11]. Increased circulating miR-1 was not associated with age, gender, blood pressure, diabetes mellitus and biomarkers for AMI. In Cheng et al., miR-1 was positively correlated with CK-MB levels [12].

Wang et al. demonstrated a significantly increased miR-133a and miR-328 levels in AMI patients and miR-133 or miR-328 levels correlated with CTnI [6]. However, the miR-133 and miR-328 exhibited faster peaks than CTnI. There were no statistical differences between the control subjects and the AMI patients for any of the considered variables except for total cholesterol (TC) and low-density lipoprotein (LDL) levels, which were elevated in patients with AMI.

Widera et al. observed that the levels of miR-1, miR-133a/b, miR-208a/b, and miR-499 were up-regulated and correlated CTnT [13]. miR-133a and miR-208b were associated with all-cause mortality at six months, even after adjustment for age and sex, but lost their association with the outcome when adjusted for hs-CTnT, indicating that these miRNAs do not add prognostic information to a sensitive myonecrosis marker.

Kuwabara et al. showed up-regulation of miR-1 and miR-133a and positively correlated with CTnT [14]. Serum levels of miR-1 and miR-133a were also increased with UA and Takotsubo cardiomyopathy without elevation of serum CK or cardiac troponins.

Gidlof et al. demonstrated the up-regulation of miR-1, miR-133a, miR-208b, miR-4995p, and miR-208b, which positively correlated with CTnT and negatively correlated with EF while other miRNAs did not correlate with EF or CTnT [15]. Although MI patients could be discriminated from non-MI patients based on the plasma levels of miR-208b and miR-499-5p, the accuracy was well below that of the current gold standard cardiac marker, CTnT. 

Devaux et al. showed that both miR-208b and miR-499 were higher in MI patients; miRNAs are present in the plasma as early as one hour after the onset of chest pain [16]. Both miRNAs correlated well with the peak concentration of CK and CTnT. However, there was also an inverse correlation with EF, indicating that miRNAs may also provide information about prognosis, but it only provided a modest prognosis of left ventricular dysfunction.

Olivieri et al. revealed that levels of miR-1, miR-21, miR-133a, miR-423-5p, and miR-499-5p were increased in NSTEMI patients vs. control. miR-499-5p and miR-21 also showed a significantly increased expression in patients with NSTEMI vs. CHF [17]. Interestingly, mir-499-5p was comparable to CTnT in discriminating NSTEMI vs. control and CHF patients. Its diagnostic accuracy was higher than conventional and hs-cTnT in differentiating NSTEMI vs. control. No significant effect of type-2 diabetes mellitus and systemic arterial hypertension was found on miR-499-5p expression levels.

Oerlemans et al. determined the potential value of circulating miR-1, miR-21, miR-146a, miR-208a, and miR-499 in a cohort of 332 suspected ACS patients, and found that the combination of miR-1, miR-21, and miR-499 could have a higher diagnostic value than hs-CTnT [18]. Multivariate logistic regression was used to investigate miRNAs independent predictability of ACS after adjustment for relevant covariates, including patient history (age, sex, previous MI, percutaneous intervention or surgery) and cardiovascular risk factors (hypertension, hypercholesterolemia, family history, current and former smoking. and diabetes mellitus).

Li et al. determined serum levels of six miRNAs in AMI patients (miR-1, -134, -186, -208, -223 and -499) up-regulated in AMI patients compared to control subjects [19]. The predictive value of measuring all six miRNA assays is better than the individual assessment of a single miRNA for the diagnosis of AMI. The miRNAs have the potential to be used complementary to the cardiac troponins for the early and accurate diagnosis of AMI. In this study, all six miRNAs presented statistically significant differences between the AMI and AP. Besides, miR-208 and miR-499 were elevated higher in AP than in AMI cases, which suggests that the two miRNAs may have a higher sensitivity in the diagnosis of AP. There was no significant difference in the age, gender, and ethnicity between the patients and the controls.

Gidlof et al. showed that the levels of all three cardio-enriched miRNAs (miR-1, miR-208a, and miR-499-5p) increased in NSTEMI patients compared to non-MI and increased amounts in STEMI patients compared to NSTEMI; however, the accuracy was well below that of the current gold standard cardiac marker, CTnT [20]. In multivariate analysis, the level of statistical significance for all correlations was unaffected by adjustment for age, sex, and sampling time.

Li YQ et al. found that the levels of miRNA-1,-133a,-208b, and -499 were significantly increased in patients after AMI compared to healthy volunteers who were matched for age and sex [21]. Although there were positive relationships between the four circulating miRNAs and cTnT within 12 hours of the onset of symptoms, none of the four circulating miRNAs was superior to CTnT for the early diagnosis of AMI.

Cheng et al. found that serum level of miR-1 increased rapidly within hours after AMI [12]. There was an over 20-fold increase in miR-1 serum level within 24 hours of AMI. Besides, there was a positive relationship between serum miR-1 and CK-MB. The result also suggested that serum miR-1 may also be related to myocardial infarct size in humans.

Chen et al. showed that plasma miR-499 levels significantly increase in the AMI group within 12 hours after the onset of symptoms and positively correlate with cardiac biomarkers [22]. It was noteworthy to find that the level of miR-499 in two- and three-vessel CAD was significantly higher than that in single-vessel CAD; thus, it positively correlates with the severity of coronary stenosis. It was also demonstrated that miR-499 levels in AMI patients 24 hours after an emergency percutaneous intervention (PCI) were significantly lower than those at admission and in the non-PCI group.

Zhao et al. revealed that miR-499 levels in AMI patients were significantly higher than those in control, but its sensitivity and specificity still lower than those of CTnI [23]. The plasma half-life of miR-499 is short; therefore, its levels significantly increase within three hours of the onset of AMI, reach a peak value at 12 hours, and then decline gradually. We expect that this phenomenon would be helpful in the diagnosis of re-infarction after an initial AMI. There was no significant difference in age and gender between the two groups.

Bialek et al. showed that miR-208a, which is produced exclusively in the heart, increases in STEMI and/or reperfusion-induced myocardial injury [24]. At the time of admission (<3 hours of the onset of symptoms), miR-208a in plasma was increased (10-fold increase) in STEMI patients when CTnI was not yet elevated. miRNA-208a also achieved its peak before both CTnI and CK-MB mass, and it shows a good correlation with the classic biomarkers of myocardial damage.

Agiannitopoulos et al. recorded the up-regulation of miR-208b and miR-499 in Greek AMI patients [25]. Blood samples were collected immediately after the patient’s admission to the hospital. It is interesting to note that Greek AMI population results are concurrent with results from Asian populations, suggesting that miRNA-208b and miRNA-499 expression levels have not been affected by the genetic background. There were no significant differences between the two groups, concerning age, gender, smoking status, and the other clinicopathological features.

Limitations

Certain limitations came across while writing this article. Firstly, not all study cohorts were age and sex-matched or matched with other cofounders. Secondly, the population size of different trials was small. Thirdly, the timing of the blood sample was not mentioned in many studies, which are very important in the case of AMI to label microRNA as an effective cardiac-biomarker for the early diagnosis of AMI. Fourthly, the levels of microRNA should be analyzed in patients with CKD to exclude any false-positive results. Large randomized cohort studies are needed to be carried to address these limitations and evaluate the potential strength of microRNAs as the new cardiac-biomarker.

## Conclusions

Our article summarizes the available literature illustrating the significance of cardio-specific microRNAs (miR-1,-133,-208, and -499) in the timely diagnosis and prognosis of the patients with AMI. We have also mentioned the correlation of these cardio-specific microRNAs with the traditional cardiac biomarkers and the time at which their levels increase in the blood. Our article is extensive as it contains studies from various parts of the world, including China, Sweden, Germany, Italy, Netherlands, Poland, Luxembourg, Japan, and Greece, maintaining that genetic and ethnic background doesn’t affect the results. Our study also highlighted that the levels of specific miRNAs might increase earlier than the traditional cardiac biomarkers in AMI. Further research at a larger scale is needed to evaluate the emerging role of microRNA in ACS as the new cardiac biomarker and to delineate their role in improving the diagnostic approach towards patients with ACS.
